# Effects of a True Prophylactic Treatment on Hippocampal and Amygdala Synaptic Plasticity and Gene Expression in a Rodent Chronic Stress Model of Social Defeat

**DOI:** 10.3390/ijms241311193

**Published:** 2023-07-07

**Authors:** Eric T. Winzenried, Anna C. Everett, Erin R. Saito, Roxanne M. Miller, Taylor Johnson, Eliza Neal, Zachary Boyce, Calvin Smith, Chloe Jensen, Spencer Kimball, Adam Brantley, Gabriel Melendez, Devin Moffat, Erin Davis, Lyndsey Aponik, Tyler Crofts, Bryson Dabney, Jeffrey G. Edwards

**Affiliations:** 1 Neuroscience Center, Brigham Young University, Provo, UT 84602, USA; 2 Department of Cell Biology and Physiology, Brigham Young University, Provo, UT 84602, USA

**Keywords:** LTP, long-term potentiation, post-traumatic stress disorder, PTSD, anxiety, rat, ventral hippocampus, basolateral amygdala, prophylactic treatment

## Abstract

Post-traumatic stress disorder (PTSD) is a complex stress-related disorder induced by exposure to traumatic stress that is characterized by symptoms of re-experiencing, avoidance, and hyper-arousal. While it is widely accepted that brain regions involved in emotional regulation and memory—e.g., the amygdala and hippocampus—are dysregulated in PTSD, the pathophysiology of the disorder is not well defined and therefore, pharmacological interventions are extremely limited. Because stress hormones norepinephrine and cortisol (corticosterone in rats) are heavily implicated in the disorder, we explored whether preemptively and systemically antagonizing β-adrenergic and glucocorticoid receptors with propranolol and mifepristone are sufficient to mitigate pathological changes in synaptic plasticity, gene expression, and anxiety induced by a modified social defeat (SD) stress protocol. Young adult, male Sprague Dawley rats were initially pre-screened for anxiety. The rats were then exposed to SD and chronic light stress to induce anxiety-like symptoms. Drug-treated rats were administered propranolol and mifepristone injections prior to and continuing throughout SD stress. Using competitive ELISAs on plasma, field electrophysiology at CA1 of the ventral hippocampus (VH) and the basolateral amygdala (BLA), quantitative RT-PCR, and behavior assays, we demonstrate that our SD stress increased anxiety-like behavior, elevated long-term potentiation (LTP) in the VH and BLA, and altered the expression of mineralocorticoid, glucocorticoid, and glutamate receptors. These measures largely reverted to control levels with the administration of propranolol and mifepristone. Our findings indicate that SD stress increases LTP in the VH and BLA and that prophylactic treatment with propranolol and mifepristone may have the potential in mitigating these and other stress-induced effects.

## 1. Introduction

According to the Diagnostic and Statistical Manual of Mental Disorders, 5th edition, text revision (DSM-5-TR), trauma- and stress-related disorders are a group of complex medical conditions. These disorders are induced by exposure to events involving traumatic or prolonged stress at any time in a person’s life and display a mix of dissociative, anxious, and depressive features. Many conditions fall under this category of psychiatric disorder, but post-traumatic stress disorder (PTSD) tends to be the most debilitating due to its long-lasting effects that can severely impair quality of life. PTSD is largely characterized by symptoms of re-experiencing (i.e., flashbacks, nightmares), avoidance, hyper-arousal, discontinuity of normal consciousness, and changes in mood and cognition [1,2,3]. Although PTSD is now a widely acknowledged condition, the underlying neural mechanisms and reasons why only a subpopulation of individuals exposed to traumatic stress develop the disorder are not fully understood. Thus, pharmacological interventions to treat the disorder are limited. However, hormones that are elevated in response to stressful stimuli—epinephrine, norepinephrine, and cortisol—are heavily implicated in disease etiology. Additionally, brain regions involved in fear conditioning, including the basolateral amygdala (BLA) and ventral hippocampus (VH), are highly dysregulated and have been implicated in PTSD [4]. The BLA receives dense innervation from the VH and is responsible for regulating fear responses [5]. These connections occur via glutamatergic neurons, which have the potential to undergo synaptic plasticity [6]. Therefore, alterations in synaptic strength due to traumatic stress within one region are likely to affect the other. 

Long-lasting synaptic changes constitute the cellular correlates of learning and memory formation and consolidation in humans and rodents [7,8], and include long-term potentiation (LTP), the activity-dependent strengthening of synapses. Dysregulated LTP has been demonstrated and is likely involved in the mechanisms of hyper-arousal and dysfunctional fear learning in rodent models of stress. Stress is a common factor that can either increase or decrease LTP, depending on the location of the recording [9,10]. For example, rodents exhibit dysregulated plasticity in the BLA and VH following chronic stress or fear conditioning [11,12,13,14,15,16]. These findings suggest a potential neurophysiological mechanism by which enhanced LTP in the BLA and VH underlies the behavioral changes observed during PTSD. PTSD-induced changes in human brain are also noted using imaging [17].

Exposure to stressful stimuli induce the production and release of stress hormones. These include glucocorticoids and catecholamines—epinephrine and norepinephrine—from the adrenal glands and norepinephrine released directly in the brain. Both corticosteroid (mineralocorticoid and glucocorticoid) and adrenergic receptors are expressed in the BLA and VH [18,19], rendering these brain areas susceptible to stress-related effects through corticosterone and norepinephrine-induced alterations of synaptic plasticity [5]. Indeed, juvenile stress increases not only LTP, but also expression of β1 adrenergic receptors and sensitivity to β-adrenergic receptors in the ventral, but not dorsal hippocampus [20]. Together, this suggests that the BLA and VH are sensitive to stress hormone signaling and that this signaling likely contributes to stress and PTSD pathology.

β-adrenergic activation in the amygdala also increases the consolidation of fear memories, inhibits fear extinction, and may provide a mechanism whereby memories of trauma pathologically persist over time [21]. Retrieval of established fear memories triggers reconsolidation processes, in which memories may be strengthened or weakened. Intra-amygdala infusion of isoproterenol, a β-adrenergic agonist, and propranolol, a β-adrenergic antagonist, respectively, strengthens and weakens fear responses [21]. Additionally, systemic administration of mifepristone, a glucocorticoid receptor (GR) antagonist, following fear conditioning reduces reconsolidation [22]. This suggests that dysfunctional fear responses in PTSD may be alterable via pharmacological intervention by inhibiting the effects of stress hormones on synaptic plasticity. However, whether prophylactic treatment with a GR antagonist or β-adrenergic antagonist can prevent the onset of PTSD symptoms remains unclear. We anticipate the incubation period of these drugs to be essential as mifepristone treatment to single prolonged stress, given 1–7 days following stress, reverses stress-induced behavior changes and sequelae, while administration 8–14 days after does not [23].

GR antagonists employed with SD stress block stress-induced activation of the ventral hippocampus [24], prevent anxiety-like behavior [25], and improve chronic stress susceptibility [26] when applied after stress. Likewise, antagonist application prior to SD blocks social avoidance and the suppression of neurogenesis associated with it [27]. In addition, the β-adrenergic antagonist propranolol reduces SD stress-induced fear conditioning activity in the BLA [28]. This illustrates the potential for GR and β-adrenergic antagonists to reduce SD stress sequelae, though their full impact on synaptic plasticity, etc. requires a full investigation.

True PTSD expression in rodents lack confirmation, so we examined a social defeat (SD) stress model as a correlate to this in rats, which has been shown to alter anxiety behavior [29]. First, we explore whether chronic SD stress is sufficient to alter VH and BLA LTP, gene expression, fear conditioning [25,28], and anxiety-like behavior [25,29]. The elevated plus maze (EPM) and light-dark transition box (LDT), two of the most commonly used behavioral tests of this nature, are used to assess approach–avoidance conflict and anxiety-like behavior in rodents. Both EPM and LDT are often used in conjunction with social defeat stress models when examining the hippocampus [30,31,32]. We then investigate whether systemic prophylactic treatment with propranolol and mifepristone is sufficient to reduce pathological changes induced by SD stress. While many prophylactic-type treatments are given soon after stress onset, we examined a true prophylactic paradigm in which treatment begins before the stress paradigm begins. This prophylactic treatment is particularly relevant to military personnel and first responders, where potential sources of trauma are more common and predictable. We determine that chronic SD stress increases LTP in the VH and BLA and that prophylactic treatment with propranolol and mifepristone may have the potential in attenuating these and other stress-induced effects.

## 2. Results 

### 2.1. Male and Female Singled Prolonged Stress (SPS) 

**Figure 1:**
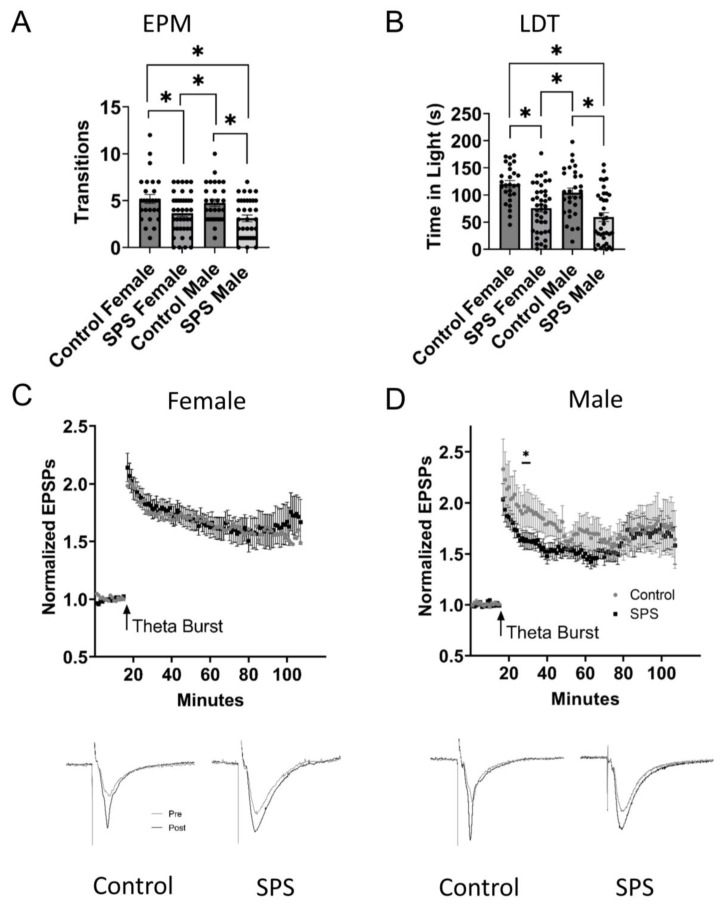
Male and female single prolonged stress (SPS) data. Comparison of elevated plus maze (EPM), light–dark transition (LDT), and hippocampal long-term potentiation (LTP) of males and females that went through the SPS protocol compared to controls. (**A**) Results from the EPM showing that SPS females and males entered into open arms significantly fewer times than controls, indicating the SPS increased anxiety-like behavior. (**B**) Results of the LDT showing that SPS females and males spent significantly less time in the light when compared to controls, again demonstrating increased anxiety-like behavior. (**C**) Hippocampal LTP levels were not different between SPS females and controls. (**D**) Hippocampal LTP levels were significantly lower in SPS males compared to controls in only the 25–29 min time interval. Insets: EPSPs are an average of 12–15 traces taken during baseline (gray) or post-theta burst conditioning (black). * Indicates significance (*p* < 0.05).

First, to identify an effective protocol for inducing chronic stress in Sprague Dawley rats, we examined two widely established stress models—single-prolonged stress (SPS) in both males and females, and social defeat (SD) in males. SPS induced significant changes in responses in both EPM and LDT in males (SPS, n = 35; control, n = 29) and females (SPS, n = 40; control, n = 27). Both sexes exhibited significantly increased anxiety-like behavior, exhibited by making fewer transitions to the open arms on the EPM (one-way ANOVA, F_3, 127_ = 6.052, *p* = 0.007; Tukey post hoc test; female SPS vs. control, *p* = 0.0089; male SPS vs. control, *p* = 0.0024) and spending less time in the light during the LDT (one-way ANOVA, F_3, 127_ = 12.32, *p* < 0.0001; Tukey post hoc test; female SPS vs. control, *p <* 0.0001; male SPS vs. control, *p =* 0.0003) when compared to controls (Figure 1A,B). A two-way ANOVA accounting for SPS treatment across three time intervals (25–29, 45–49, 85–89 min) revealed a significant effect of SPS treatment on hippocampal LTP in male rats (SPS, n = 18; control, n = 25; F_1, 525_ = 11.38, *p* = 0.0008). No effect was observed in female rats (SPS, n = 28; control, n = 18; F_1, 591_ = 0.3719, *p* = 0.5422). Bonferroni post hoc tests revealed that SPS significantly reduced LTP in male rats at 25–29 min (*p =* 0.0023) but not at 45–49 min (*p* = 0.0552) or 85–89 min (*p* = 0.9999). In summary, while the SPS protocol induced significant stress, no significant differences were noted in female ventral hippocampal LTP when compared to controls, and only a small change in one time window for males (Figure 1C,D). As a result, the remainder of our research focused on SD, which induced significant changes in behavior, physiology, mRNA expression, and plasma hormone levels between control, SD, SD vehicle-treated (SDV), and SD drug-treated (SDD) rats. Although not included in the final analyses, the SPS data are included here for a full assessment of methodologies to ensure a comprehensive description of the current study, as well as reproducibility and rigor for those doing future studies. 

### 2.2. Ventral Hippocampal and Basolateral Amygdala Field Electrophysiology 

**Figure 2:**
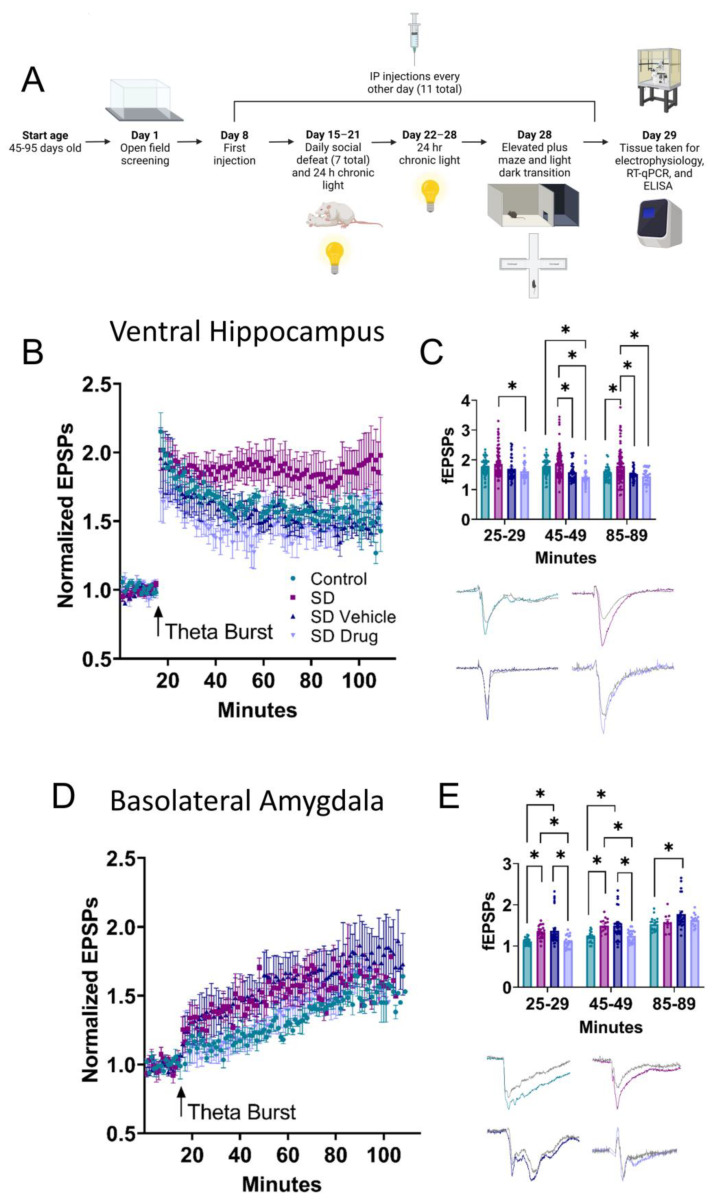
Hippocampal and basolateral amygdala (BLA) field electrophysiology. (**A**) Timeline of the methods and experiments. (**B**) Field EPSPs (fEPSPs) were measured following theta burst (arrow) to induce long-term potentiation (LTP) in the hippocampus in control, SD, SDV, and SDD rats. (**C**) The LTP was measured as percent increase compared to the hippocampus and significant differences were noted between some of these groups as measured at varying time points. (**D**) LTP was also measured in the BLA in control, SD, SDV, and SDD rats. (**E**) LTP was measured as percent increase compared to baseline in the BLA, as visualized in bar graphs to demonstrate significance. Insets: Representative traces are an average of 10–15 sweeps taken during baseline (dark grey) or 45–49 min post-conditioning (group color). All measurements taken were from monosynaptic events and excluded the fiber volley. * Indicates significance (*p* < 0.05).

Using the SD model, field electrophysiology experiments were performed in the CA1 VH and the BLA to determine changes in LTP associated with stress conditioning and drug treatment. In the CA1, a two-way ANOVA revealed significant differences in LTP between control (n = 13), SD (n = 20), SDV (n = 8), and SDD (n = 7) rats that were further compared across three different time intervals: 25–29, 45–49, and 85–89 min (F_3, 631_ = 26.77, *p* < 0.0001) (Figure 2B,C). Tukey post hoc analysis revealed significantly higher LTP in SD rats compared to control rats at 85–89 min (*p* = 0.0014) and compared to SDD rats at every time interval (25–29, *p* = 0.0034; 45–49, *p* < 0.0001; 85–89, *p* < 0.0001). SD rats also displayed significantly higher LTP than SDV rats at 45–49 (*p* = 0.0002) and 85–89 min (*p* = 0.0010). LTP was significantly lower in SDD compared to control rats at 45–49 min (*p* = 0.0006). These results suggest that prophylactic drug treatment significantly reduced SD-induced enhancements in LTP. 

In the BLA, two-way ANOVA also revealed significant differences in LTP in control (n = 9), SD (n = 9), SDV (n = 13), and SDD (n = 12) rats (F_3, 235_ = 16.14, *p* < 0.0001) (Figure 2D,E), which were compared across the same three time intervals. Tukey post hoc analysis showed that the SD and SDV rats displayed significantly higher LTP than the control at 25–29 (SD vs. control, *p* = 0.0235; SDV vs. control, *p* = 0.0019) and 45–49 min (SD vs. control, *p* = 0.0055; SDV vs. control, *p* = 0.0005). At 85–59 min, SDV rats also displayed significantly elevated LTP compared to control rats (*p* = 0.0080). SD and SDV rats had significantly elevated LTP compared to SDD rats at 25–29 (SD vs. SDD, *p* = 0.0191; SDV vs. SDD, *p* = 0.0015) and 45–49 min (SD vs. SDD, *p* = 0.0196; SDV vs. SDD, *p* = 0.0031). No significant differences in LTP were observed between control and SDD rats in any time interval (25–29, *p* = 0.9999; 45–49, *p* = 0.9722; 85–89, *p* = 0.6731). Additionally, there were no significant differences between SD and SDV rats (25–29, *p* = 0.8969; 45–49, *p* = 0.9999; 85–89, *p* = 0.1134). At 85–89 min, no significant difference was observed between SD and control (*p* = 0.9802), SD and SDD (*p* = 0.9316), and SDV and SDD (*p* = 0.2880). Together, these data indicated that SD enhanced LTP in the VH and BLA, while prophylactic drug treatment generally reversed these effects. 

### 2.3. Behavioral Testing in the Elevated plus Maze and Light-Dark Transition Test

**Figure 3:**
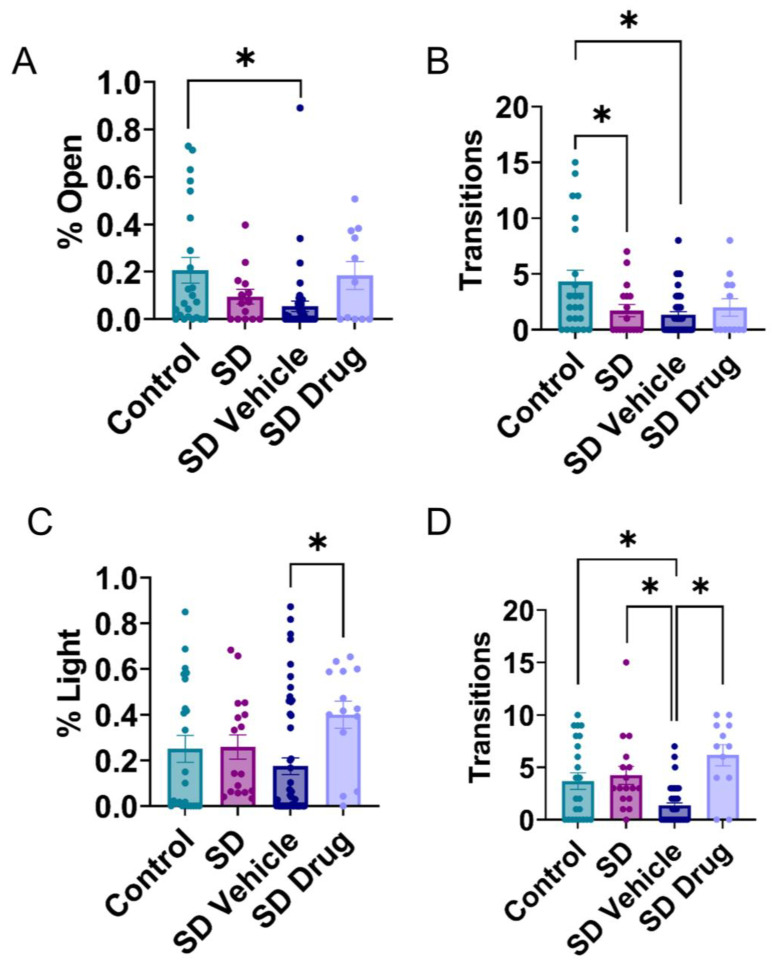
Behavioral testing in the elevated plus maze and light–dark transition test. In the elevated plus maze, the proportion of time rats spent in the open arms (**A**) and number of transitions into the open arms (**B**) were measured in control, SD, SDV, SDD treated rats. The percent time spent in the light (**C**) was recorded in control, SD, SDV, SDD treated rats. The number of transitions into the light (**D**) was recorded in control, SD, SDV, SDD treated rats.* Indicates significance (*p* < 0.05).

Behavioral assays were conducted using the elevated plus maze (EPM) and light–dark transition (LDT) the day before each rat was sacrificed to determine changes in behavioral anxiety and avoidance. A one-way ANOVA indicated significant differences in relative amount of time control (n = 23), SD (n = 17), SDV (n = 51), and SDD (n = 12) treated rats spent in the open arms of the EPM (F_3, 89_ = 4.202, *p* = 0.0079). Tukey’s post hoc analyses showed that SDV rats spent significantly less time in the open arms (*p* = 0.0083), while SDD rats displayed no significant difference compared to the control rats in the EPM (*p* = 0.9872) (Figure 3A). Furthermore, a one-way ANOVA indicated significant differences in the number of transitions from closed to open arms of the maze (F_3, 99_ = 5.305, *p* = 0.002). Tukey’s post hoc test revealed that SD (*p* = 0.0388) and SDV (*p* = 0.0009) rats made significantly fewer transitions to open arms than the control rats (Figure 3B). In the LDT assay, one-way ANOVAs revealed significant differences in control (n = 23), SD (n = 17), SDV (n = 51), and SDD (n = 15) treated rats for time spent in the light compartment (F_3, 101_ = 2.981, *p* = 0.0349) and the number of transitions into the light between treatment groups (F_3, 99_ = 12.088, *p* < 0.001). Tukey’s post hoc test revealed that SDD rats spent significantly more time in the light compartment compared to SDV rats (*p* = 0.0216) (Figure 3C). Tukey’s post hoc test also indicated no significant difference between the control and SD rats in the number of transitions into or time spent in the light compartment (*p* = 0.93) (Figure 3D). However, SDV rats transitioned significantly less into the light compartment compared to the control (*p* = 0.008), SD (*p* = 0.003), and SDD (*p* < 0.001) (Figure 3D). These data indicate that in some measures, our SD paradigm induced behavioral anxiety. However, in other measures, no effect was observed. Additionally, there may be an anxiety-inducing injection effect, as there was a significant difference between SD and SDV in LDT transitions.

### 2.4. Plasma Corticosterone, Norepinephrine, and ACTH Concentrations

**Figure 4:**
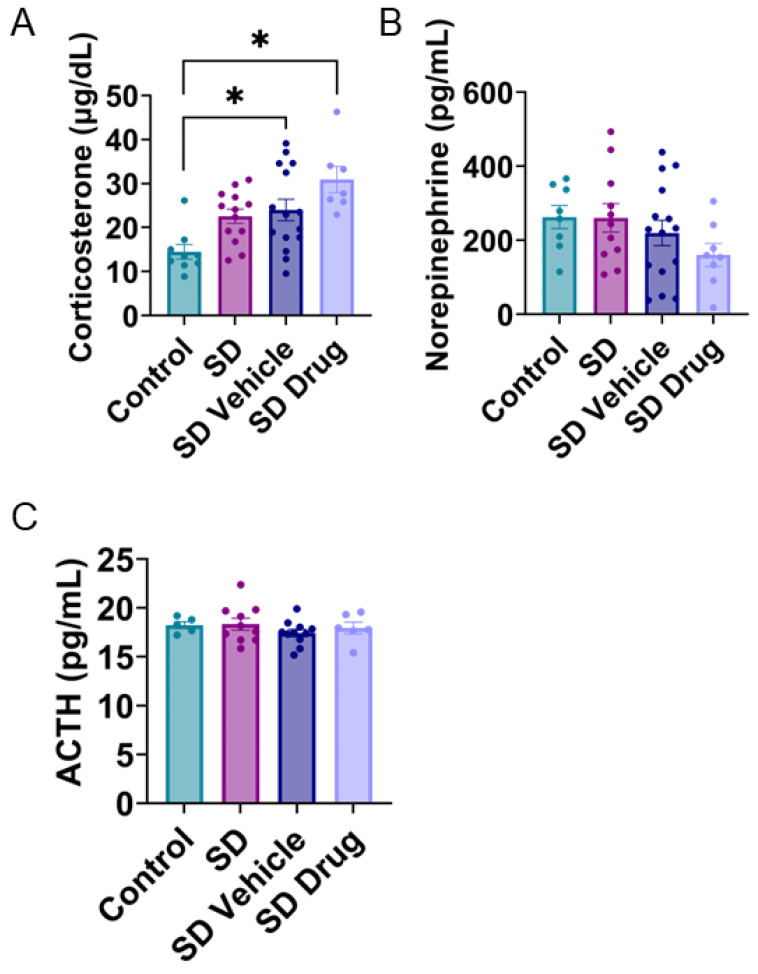
Plasma corticosterone, norepinephrine, and ACTH concentrations. Corticosterone (**A**), norepinephrine (**B**), and ACTH (**C**) levels measured from control, SD-, SDV-, and SDD-treated rats. * Indicates significance (*p <* 0.05).

Competitive ELISAs were performed to determine concentrations of plasma corticosterone, norepinephrine, and adrenocorticotropic hormone (ACTH), which are commonly altered following chronic stress in both human and rodent models. One-way ANOVA indicated significant differences in plasma corticosterone concentrations (control, n = 9; SD, n = 13; SDV, n = 15; SDD, n = 7; F_3, 40_ = 6.640, *p* < 0.001) (Figure 4A). Tukey’s post hoc test indicated that SDD (*p* < 0.001) and SDV (*p* = 0.021) rats both demonstrated significantly higher concentrations of corticosterone compared to control rats. Although there were no significant differences outside of these two comparisons, it is worthwhile to note that SD-treated rats compared to controls displayed trends toward significance (*p* = 0.075) (Figure 4A), and SD and SD-vehicle were not significantly different from each other. One-way ANOVA indicated no significant differences between any treatment groups in plasma norepinephrine (control, n = 8; SD, n = 11; SDV, n = 15; SDD, n = 8; F_3, 38_ = 1.449, *p* = 0.244) (Figure 4B) nor ACTH levels (control, n = 5; SD, n = 11; SDV, n = 11; SDD, n = 6; F_3, 28_ = 0.7224, *p* = 0.547) (Figure 4C). 

### 2.5. Relative mRNA Expression Levels of Post-Chronic Stress-Related Target Genes 

To investigate mechanisms of prophylactic treatment on gene expression, quantitative RT-qPCR (qPCR) was used to quantify changes in mRNA in VH and BLA homogenates. We examined genes involved in excitatory synapse plasticity, stress hormone signaling, and epigenetic modification. Together, significant changes in mRNA expression were observed in glucocorticoid receptors (GR), mineralocorticoid receptors (MR), glutamate α-amino-3-hydroxy-5-methyl-4-isoxazolepropionic acid receptor 1 (AMPA1), AMPA receptor 2 (AMPA2), glutamate N-methyl-D-aspartate receptor 2A (NMDA2A), period circadian regulator 1 (PER1), and CREB binding protein (CREBBP). See Table 1 for a full list of gene targets.

**Figure 5:**
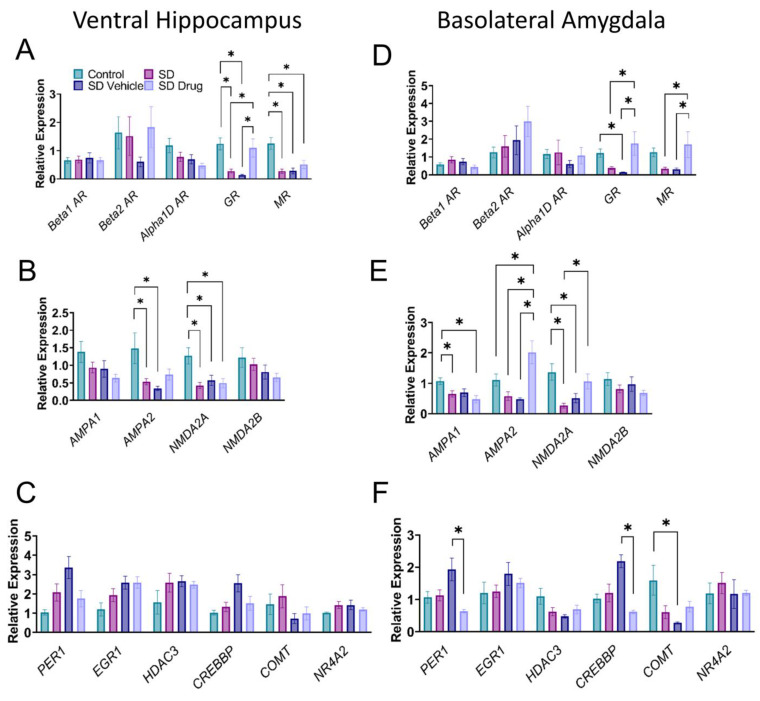
Relative mRNA expression levels of post-chronic stress-related target genes. Expression levels of (**A**) VH and (**D**) BLA stress hormone-related genes (**B**) VH and (**E**) BLA plasticity-related genes (**C**) VH and (**F**) BLA signaling and epigenetic modifier genes were determined from tissue samples from control, SD, SDV, SDD rats. * Indicates significance (*p <* 0.05).

In hippocampal homogenates, one-way ANOVA revealed significant changes in gene expression between groups (Figure 5A,B) for GR (control, n = 11; SD, n = 14; SDV, n = 11; SDD, n = 13; F_3, 45_ = 7.72, *p* = 0.0003), MR (control, n = 8; SD, n = 17; SDV, n = 11; SDD, n = 13; F_3, 45_ = 10.59, *p* < 0.0001), AMPA2 (control, n = 12; SD, n = 17; SDV, n = 11; SDD, n = 13; F_3, 49_ = 4.663, *p* = 0.0061), and NMDA2A (control, n = 10; SD, n = 16; SDV, n = 11; SDD, n = 13; F_3, 46_ = 6.435, *p* = 0.001). Tukey post hoc analysis revealed that of the stress-related receptors, GR and MR expression was significantly reduced in SD (GR, *p* = 0.0069; MR, *p* < 0.0001) and SDV (GR, *p* = 0.0029; MR, *p* < 0.0001) rats compared to control rats (Figure 5A). Interestingly, while drug exposure (SDD) did not significantly increase GR compared to controls (*p* = 0.9543), SDD was significantly different from SD (*p* = 0.0200) and SDV (*p* = 0.0082), demonstrating a rescuing effect of drug treatment. However, this rescuing effect was not observed in MR expression, which was significantly lower in SDD rats compared to control rats (*p* = 0.0022). No difference was noted in adrenergic receptors. In the plasticity-related genes, AMPA2 expression for SD (*p* = 0.0152) and SDV (*p* = 0.0073) were significantly different from controls, but SDD was not (*p* = 0.1099). In addition, NMDA2A expression was significantly reduced in SD rats compared to control rats (*p* = 0.0009) but was not rescued with drug treatment, as expression was also significantly lower in SDD rats compared to control rats (*p* = 0.0043) (Figure 5B). There were no significant differences in the epigenetic modifiers between any groups (Figure 5C). 

One-way ANOVA also revealed significant changes in BLA mRNA expression between treatment groups (Figure 5D,F) for GR (control, n = 11; SD, n = 14; SDV, n = 10; SDD, n = 7; F_3, 38_ = 7.653, *p* = 0.0004), MR (control, n = 8; SD, n = 14; SDV, n = 10; SDD, n = 7; F_3, 35_ = 5.421, *p* = 0.0036), AMPA1 (control, n = 11; SD, n = 14; SDV, n = 10; SDD, n = 7; F_3, 38_ = 4.519, *p* = 0.0083), AMPA2 (control, n = 9; SD, n = 14; SDV, n = 10; SDD, n = 7; F_3, 36_ = 11.65, *p* < 0.0001), NMDA2A (control, n = 10; SD, n = 14; SDV, n = 10; SDD, n = 7; F_3, 37_ = 8.157, *p* = 0.0003), PER1 (control, n = 5; SD, n = 14; SDV, n = 4; SDD, n = 8; F_3, 26_ = 4.9, *p* = 0.0079), and CREBBP (control, n = 5; SD, n = 14; SDV, n = 4; SDD, n = 7; F_3, 26_ = 3.816, *p* = 0.0217). Tukey post hoc analysis indicated, similar to the hippocampus, that SDD drug exposure significantly altered GR levels compared to SD (*p* = 0.0038) and SDV (*p* = 0.0013) but not controls (*p* = 0.5019). There was also a trend toward downregulation of MR expression in SDV rats compared to controls (*p* = 0.1238) and a significant upregulation in SDD rats compared to both SD (*p* = 0.0107) and SDV (*p* = 0.0152) rats (Figure 5D). SDD MR expression was not significantly different compared to controls (*p* = 0.7763), illustrating a drug-mediated return to control levels. In plasticity-related genes, AMPA1 was significantly lower in SDD compared to control rats (*p* = 0.0091) (Figure 5E). Conversely, AMPA2 expression was significantly higher in SDD rats compared to all other groups (control, *p* = 0.0217; SD, *p* < 0.0001; SDV, *p* < 0.0001) and NMDA2A expression was significantly lower in SD compared to control rats (*p* = 0.0003). In the epigenetic regulators that were assessed, expression was significantly lower in SDD compared to SDV rats for both PER1 (*p* = 0.0038) and CREBBP (*p* = 0.0124), but not statistically different from control rats (*p* = 0.7776) (Figure 5F). These changes in epigenetic regulators were specific to the BLA, as we did not observe similar changes in the hippocampus. Overall, these data revealed a generally restorative effect of drug treatment on the expression of both stress hormone and plasticity-related genes in the hippocampus and BLA following SD exposure. 

## 3. Discussion 

Here, we demonstrate that rats selected for a predisposition to anxiety-like behavior and exposed to our chronic SD-based stress protocol (SD combined with social isolation and chronic light) display increased transitions in the LDT and EPM. The typical anxiety-related readout of the EPM, time in open arms, was only reduced when the chronic SD stress is preceded by a vehicle injection as well. In this model, we also note changes in anxiety-like behavior, stress hormones, hippocampal and amygdala LTP, and gene expression. These effects were largely reversed with the systemic prophylactic administration of propranolol and mifepristone (β-adrenergic receptor antagonist and glucocorticoid receptor antagonist, respectively), indicating these stress-induced changes in behavior and brain physiology following chronic SD stress were likely dependent on norepinephrine and/or glucocorticoid hormones and their downstream effects. Together, these data suggest propranolol and mifepristone as potential therapeutics for preventatively mitigating the negative effects of stress on anxiety, as well as plasticity and gene expression in the VH and BLA. 

To study the cellular effects of traumatic stress on LTP in the brain regions implicated in PTSD, a rodent model was adopted. While rodents share many of the same biological and behavioral characteristics of PTSD as humans, current models are imperfect in replicating all PTSD pathologies [33,34,35,36]. To ensure the most accurate model possible, it is recommended to test multiple models and assess their efficacy in producing the pathology of interest. Both SPS and SD have demonstrated PTSD-like behavioral outcomes [35]. Because of this, both SPS and SD models were initially investigated and included in the presented data here. Along with the basic model of SPS and SD, the rats were exposed to a stressor of two weeks of chronic light, which has also been shown to demonstrate stress-like behavior in rodents [37]. Even though both SPS and SD increased anxious-like behaviors compared to controls, the SPS protocol did not induce significant changes in ventral hippocampal LTP in rats. In contrast, the SD stress protocol showed a significant increase in both VH and BLA LTP compared to controls. Therefore, the SD model was chosen for the study. 

As previously indicated, the fact that only 60–70% of animals exposed to extreme stress display PTSD-like symptoms poses a large hurdle for developing animal PTSD models and PTSD research, at large [34]. To account for this and increase the likelihood of inducing the desired stress effects, we screened for animals with a predisposition to anxious behavior, as resilience to stress is the exception and not the rule [38]. Prior studies have successfully employed similar methodologies and screened rodents into susceptible and resistant (non-susceptible) groups using social defeat [26,28]. While our SD model was shorter than some other, more traditional approaches [35], we adapted our methodology to produce robust stress effects [39]. With these adaptations, we observed significant changes in behavior and physiology, including increased BLA activity and plasticity [33]. 

In this study, we did not quantify the exact number of aggressive and submissive behaviors during SD interactions, which would have provided insight into whether drug-treated rats exhibited conditioned defeat behavior following repeated SD interactions. Future studies should include this type of analysis and correlate it with other experimental outcomes. Additionally, social stress research should consider not only the method/environment used but factors including controllability based on victims’ response to stress, which could explain individual vulnerability [40]. 

Rodents display two major coping styles when exposed to social defeat stress—proactive, aggressive coping or reactive, docile coping [41,42,43]. Because this distinction is also relevant to humans in predicting physical/mental health outcomes in response to stress [44,45,46], rodent social stress research often categorizes rodent data based on coping styles. After initial screening for rats with anxious predispositions, few rats displayed proactive coping and were subsequently removed from the study. The vast majority of anxious-screened rats exhibited reactive coping, similar to previous studies of rodents with anxiety-like behaviors [47]. In rodents, chewing is also a long-characterized coping mechanism for stress [48]. However, we did not note any changes in chewing activity. Sex can also affect social defeat stress behavior [49]. While we focused on males due to our model of social defeat choice, sex should still be considered as a potential variable. It is also worth noting mifepristone injections prior to social defeat stress in our experiments may have improved susceptibility to chronic stress.

The BLA is anatomically situated such that it acts as the sensory interface of fear learning, receiving projections from the hypothalamus, brain stem, cortex, etc. [15]. Studies of amygdala involvement in fear conditioning have demonstrated that synaptic plasticity of inputs to the BLA underlies fear acquisition as well as the storage of fear memories [50]. The amygdala also modulates memory consolidation in the hippocampus, which is relevant to PTSD [51]. Under non-pathological conditions, prefrontal cortex projections to the amygdala normally inhibit inappropriate fear responses. As such, PFC lesions in humans are associated with the inability to regulate emotions [52]. Although we did not explore PFC-specific mechanisms, we observed a significant increase in BLA LTP, which is consistent with this framework and our behavioral data, which revealed significant elevations in anxiety-like behavior with SD stress (see Figure 3A,D). 

PTSD is typically characterized by persistent intrusive memories, but is also associated with impairments in cognition and memory [52]. One PTSD-like rodent model has suggested that under highly emotional and stressful conditions, the hippocampus switches from cognitive mapping to processing and storing fragments of memories [53]. While the hippocampus is generally viewed as the brain’s “memory center” (as reviewed by [54]), the hippocampus can be further divided into separate structures with distinct functions—the dorsal (DH), intermediate, and ventral (VH) hippocampus. The DH primarily performs cognitive functions (e.g., spatial memory) due to its connections to the retrosplenial and anterior cingulate cortices, which are highly involved in the processing of visuospatial information. On the other hand, the VH primarily performs functions related to emotion, stress, and fear learning due to the bidirectional connections between the CA1 and subiculum, and the BLA [55]. Here, we observed significant elevations in LTP in the VH, which are indicative of enhanced fear learning and are also consistent with our behavioral data. Previous work has revealed that following acute stress, the VH experiences enhanced LTP while the DH has depressed LTP [20,56]. This increase in LTP has also been observed in the BLA during both acute [19] and chronic stress [57]. Since the BLA and VH are connected and are both involved in stress, fear, and emotion as reviewed by [58], this enhanced plasticity is invariably linked to stress-related disorders.

The elevations we observed in behavioral anxiety and LTP in both the BLA and VH were in large part prevented with the prophylactic administration of propranolol and mifepristone, implicating norepinephrine and/or corticosterone in these effects. Although we did not observe significant changes in plasma NE, we determined this was likely an effect of collecting blood seven days post-SD stress, at which time, plasma NE returned to basal levels. NE is generally thought to mediate fast-acting, acute stress effects, while HPA activation and downstream cortisol secretion are generally thought to mediate longer-term, chronic stress responses [59], which is consistent with the data presented here. 

Cortisol readily crosses the blood–brain barrier, passively enters cells, and binds to mineralocorticoid and glucocorticoid receptors (MR, GR). These induce both fast-acting, non-genomic effects as well as delayed, genomic effects on synaptic transmission via activating gene transcription [60]. The balance of MR and GR expression is necessary to maintain homeostasis and neuronal excitability such that MR enhances and GR reduces excitability as reviewed by [61]. Previous studies reported significant downregulation of MR and GR expression in both the hippocampus [62] and amygdala [63] of rats exposed to single prolonged stress. Our results are consistent with these reports in that we observed significant downregulation of GR (NR3C1) and MR (NR3C2) in response to SD stress in both the VH and BLA. We determined this to be a possible compensatory response to elevated plasma corticosterone with SD stress and may contribute to our observed elevations in LTP, as GR and MR expression largely reverted to control levels with drug administration with the exception of MR in the VH. It is important to note that while MR and GR mRNA expression was significantly lower in SD and SDV rats compared to controls in both the VH and BLA, prophylactic drug treatment largely restored receptor expression to control levels. Corticosterone, however, remained high in these groups, which suggests that prophylactic drug treatment does not decrease corticosterone to prevent SD-induced changes in the brain, but rather, blocks GR and MR to maintain control levels of expression. 

There are several caveats or alternatives to consider in the study as well. Note that while corticosterone increases in stressful situations, [64] in some studies, corticosterone has remained elevated 48 h after the last episode of stress conditioning [26], while others have observed that corticosterone returns to controls levels 48 h following SD stress [29]. The data we present here are consistent with the former study in that we observed significant changes in plasma corticosterone several days after the last episode of SD stress. While we did not measure corticosterone concentrations acutely post-SD stress, the elevations in corticosterone we observed at sacrifice suggest that these elevations persisted over the course of stress conditioning. 

Although we observed significant elevations in corticosterone with stress conditioning, it is important to note that the elevation alone does not necessarily indicate the emotional valence of the social stimulus (i.e., whether the stimulus is positive or negative). For example, one study demonstrated that social defeat stress and mating induced similar elevations in corticosterone [65], which are generally perceived as opposites in social valence. Despite this, the combination of our behavior and physiological data suggests the elevations in corticosterone were negative. 

While this could suggest mifepristone could just be blunting the HPA axis, our data illustrate significant changes in molecular biology, plasticity, and cognition when combined with propranolol. Lastly, drug treatment could block the intruder rat from having a salient experience. This could interfere with the outcomes of a stressful experience such as coping. 

The relationship between stress hormone corticosterone/cortisol and memory is well established as following an inverted-U shape, particularly in the dorsal hippocampus, such that mild levels of stress enhance, and extreme levels of stress impair hippocampal plasticity [66,67,68]. This curve uses serum corticosterone as a proxy for the level of stress. Therefore, corticosterone’s impact on hippocampal plasticity is not only dependent on DH versus VH but on concentration as well. The level of corticosterone we measured with SD treatment is considered high based on these studies. Significant elevations in corticosterone were also observed with drug treatment. Blocking GR receptors with mifepristone has been shown to also inhibit negative feedback of corticosterone leading to elevations in corticosterone and ACTH [69]. We observed a significant elevation in plasma corticosterone with the administration of mifepristone, which is consistent with this. Treatment with mifepristone and propranolol reverted LTP to control levels. While the data were not completely consistent in the VH and BLA, together they suggest that the elevations in LTP we observed with SD stress were corticosterone-dependent.

The data presented, combined with previous studies, indicate that GR and β-adrenergic antagonists may be promising potential treatments for stress-related sequelae. GR antagonists block stress-induced activation of the ventral hippocampus [24], prevent anxiety-like behavior [25], improve susceptibility to chronic stress [26], and eliminate anxiety-like behavior induced by traumatic brain injury [70]. In addition, GR antagonist application reverses stress-induced dysregulation of anxiety SPS if administered within 7 days of the stress, but not after 8 days [23], illustrating the importance of timing of drug administration. β-adrenergic antagonist propranolol has been shown to reduce SD stress-induced fear conditioning activity in the BLA [28]. 

While studies that apply antagonists before stress conditioning are limited, the few studies that do so demonstrate promise as potential prophylaxis. For example, GR antagonist application prior to SD has been shown to block SD-induced social avoidance and suppression of neurogenesis [27]. Administration of a GR antagonist before SPS has been shown to prevent impairments in LTP in the CA1 one week after stress [71]. Although the location within the hippocampus was not reported in that study, it was likely dorsal, which agrees with research from others and our lab [72] that demonstrates stress depresses LTP in the dorsal VH. Here, we examined VH, where we saw stress-induced enhancements in LTP. Propranolol and mifepristone have been shown to quickly an independently counteract the increase in BLA LTP when injected 30 min before acute stress [19]. These studies are among the few that include a “true” prophylactic administration of these drugs, as opposed to administration shortly after the stress protocol. Our study is unique in that it combines a true prophylactic treatment with chronic stress. Propranolol and mifepristone were administered in combination to maximize the likelihood of seeing significant results; further research must clarify their independent effects. Other potential targets for reversing stress-induced behavioral changes that remain to be fully examined include ketamine [26,73] as well as the BDNF-TrkB pathway [28,74]. 

In primary hippocampal neurons, it has been demonstrated that corticosterone enhances AMPA receptor mobility, such that corticosterone increases AMPA receptor trafficking to the membrane under conditions of long-term potentiation (LTP) [75]. Corticosterone also increases the surface expression of AMPA2 in rat hippocampal neurons [76]. Although we do not have data on the subcellular localization of AMPA receptor expression, our data demonstrate that SD stress significantly reduces AMPA1 mRNA expression in the BLA and AMPA2 mRNA expression in the VH and BLA. Although these changes were inconsistent with what we would have expected based on our LTP data, these reductions in gene expression were largely reversed with drug administration (see Figure 4A,C). It is possible that synaptic plasticity was enhanced via other mechanisms that are not detectable by qPCR in these stressed animals (e.g., phosphorylation of AMPA1 subunits to enhance receptor conductance, enhanced trafficking of receptors to the membrane). However, this would require further investigation. Additionally, it has been shown that norepinephrine activation of adrenergic receptors in these regions enhances the phosphorylation of the AMPA1 subunit of alpha-amino-3-hydroxy-5-methyl-4-isoxazolepropionic acid (AMPA) receptors, which upregulated receptor trafficking to the membrane, and therefore, LTP [77]. In addition, NMDA2A was also significantly altered by SD and SD vehicle control compared to naïve control, which was recovered by prophylactic drug treatment. These changes could lead to altered plasticity as well. It is also important to note that mRNA levels do not directly correlate to synaptic receptor expression levels but are suggestive only; however, qRT-PCR studies are valuable in providing potential targets for future investigation. 

Lastly, one of the biggest hurdles in producing translatable PTSD research has been the difficulty of modeling such a complex psychiatric disorder in rodents. Many currently employed rodent stress models employ SD stress types in an attempt to correlate to PTSD. In our study, we added a chronic ongoing light stressor to acute SD stress, as PTSD in humans often occurs subsequent to both chronic ongoing stressors with occasional acute stress. We did this to attempt to increase the translational relevance of our model. Additionally, we waited 7 days following chronic stress before performing behavioral and electrophysiological experiments, as PTSD symptoms typically manifest after a delayed period in humans. Our model was able to mimic many of the symptoms of stress pathology including elevated plasma corticosterone, behavioral anxiety, VH and BLA LTP, and changes in gene expression. With that said, many of the symptoms typically used to diagnose humans (e.g., flashbacks and nightmares) are undetectable in rodents. In addition, we first employed a screening method as not all humans exposed to extreme stress develop PTSD. Therefore, our model does not directly correlate to an entire population but does model a high-anxiety group. Collectively, our prophylactic treatment appears to have promising effects in our rodent chronic SD model; however, the translational relevance may be limited due to unpredictable stressors. In particular, first responders and military personnel would be the most likely to benefit from this type of treatment as trauma is more predictable. Future studies in humans should investigate the potential of mifepristone and propranolol as preventative treatments for PTSD in these groups.

## 4. Materials and Methods

### 4.1. Animals

All experiments were performed in accordance with Institutional Animal Care and Use Committee (IACUC) protocols and followed National Institute of Health guidelines for the care and use of laboratory animals. IACUC protocols for all experiments were approved by the Brigham Young University Institutional Animal Care and Use Committee. Male and female Sprague Dawley rats between the ages of 60 and 110 days were used for electrophysiology, RT-qPCR, and behavioral experiments. We observed no significant differences in any experiments accounting for age. 

### 4.2. Screening Protocol

PTSD only effects a portion of those exposed totraumatic situations [38,78]. Fifteen days prior to initiating the two-week single prolonged stress (SPS) or SD, male Sprague Dawley rats (45–95 days old) were screened to identify the individuals with the highest probability of developing pathological anxiety or stress-like symptoms. The screening employed an open-field test modeled after Nalloor et al. [79] and Wernecke et al. [80], based on earlier findings that stress susceptible rodents can be differentiated from non-susceptible groups. Rats were subject to three 10 min habituation sessions in an open-field arena without any other stimuli. The open field was divided into four quadrants. A ball of cat fur and a single drop of natural fox urine were placed in one of the quadrants. This combination of cat fur and fox urine was used as a robust means of eliciting anxious-like behavior to sort rats into anxious and non-anxious groups [80]. Rats were placed in the arena opposite of the cat fur and fox urine and observed for five minutes for anxious behavior. Anxious behavior consisted of immobility, avoiding the quadrant containing cat fur and fox urine, urination or defecation, and lack of rearing (standing on hind legs) [81,82]. Non-anxious rats did not display these traits while semi-anxious and anxious were identified based on the number of behavioral responses but were combined into one anxious group to create two final groups, anxious and control. Note that discriminating between anxious and semi-anxious was often difficult and physiology or behavioral differences were not noted within the anxious group, thus justifying combining the groups [80]. The open field arena was cleaned between each rat. 

Rats were group-housed in cages of two or three littermates until they began the stress protocol, at which point they were housed individually. We note that 66% of total rats screened were susceptible to this protocol. We used a total of 155 susceptible and 79 resistant rats for this study. Variation in the number of rats was largely due to variation in allotment of rats to groups and the success of electrophysiology experiments. For example, after behavior experiments were performed, some rats were used for hippocampal physiology while others were used for amygdala physiology experiments. Additionally, some experiments failed, which left us with successful behavioral tests but no physiological data. However, all the data included in the analyses and figures were reliable and all experiments were sufficiently powered to make interpretations. Despite the unequal sample sizes in, for example, behavioral tests, variance between groups was not unequal. Variance was assessed via visual assessment of data boxplots and calculation of variance, which differed by less than one order of magnitude between groups. Validating the assumption of equal variance allowed us to continue with the ANOVA analyses. After screening, rats were randomly divided into control, SD, SD vehicle-treated (SDV), and SD drug-treated (SDD) groups.

### 4.3. Single Prolonged Stress

Male and female Sprague Dawley rats were used for the SPS protocol. Throughout the 14-day protocol, rats were maintained in isolation and exposed to 24 h of chronic light (~5 lux). In the SPS conditioning, rats were subject to two hours of restraint in a tube manufactured by the Precision Machining Laboratory at BYU, followed immediately by a forced swim for 20 min in room temperature water. After the rats were dried off, they were held in a chamber with 2% isoflurane until a loss of consciousness, at which point, rats were then moved back to their original cages to recover. The SPS occurred on day one and day eight of the stress protocol. On day 14, behavioral tests (elevated plus maze and light–dark transition) were performed. Rats were sacrificed on day 15.

### 4.4. Social Defeat 

Male Sprague Dawley rats were used for the SD protocol. In our observation, female rats were less aggressive and did not consistently socially defeat their intruders. Therefore, females were excluded from this study, as chronic stress would have been difficult-to-impossible to induce using this technique. In SD, large male rats were used as the aggressive “defender” or “resident”. These males were bred with females occasionally to promote continued “defender”-type behavior in the SD protocol. Young, anxious-screened males were used as “intruders”. 

Intruder rats were isolated and exposed to 24 h chronic light (~5 lux) for the 14 days of the protocol. During the SD protocol, an intruder was placed in a defender’s cage and allowed to interact with the defender for 5 min. After 5 min, a wire mesh barrier was placed between the two rats in the cage for an additional 25 min. This barrier allowed the rats to see and smell each other but prevented them from physically interacting. If the defender was overly aggressive with the intruder, the rats were immediately separated by the mesh barrier to prevent injury. Although the number of aggressive and submissive behaviors was not quantified, they were observed in nearly all SD interactions. 

Because the goal of social defeat conditioning was to induce social stress in intruder rats, resident rats were retired from the social defeat protocol when they were non-aggressive and ignored the intruder rats. Intruder rats that did not experience social defeat for 4 or more days due to non-aggressive interactions during the SD protocol from the resident rat’s lack of aggression were also excluded from further experiments. Aggressive behavior was classified as aggressive posturing, biting, or chasing between the defender and intruder rats. Ten total rats were excluded from the three SD groups for this reason and no further experiments were performed. Additionally, in a few cases, intruder rats were excluded if they showed no signs of submission or intimidation (e.g., supine posture, calls of distress, freezing) despite aggressive encounters in the SD protocol. Studies have shown that rats with high anxiety-like behavior are more likely to display docile (reactive) coping strategies while rats with low anxiety-related behavior were more likely to engage in aggressive (proactive) coping [47], and thus actively coping rats were removed. Only four intruders fell into this category of active coping behaviors and were removed from further experiments. The vast majority of intruder rats, however, displayed docile coping and high anxiety-like behavior, which indicated our screening protocol was efficient. Rats that were eliminated based on these criteria were done so before behavior or physiology experiments were performed in order to prevent any biases. 

After the 30 min of SD, the intruder was returned to its original cage in isolation under chronic light. The SD protocol lasted 7 consecutive days beginning on the first day of chronic light exposure. A different defender was used each day to avoid familiarity between the defender and intruder. Following the seventh day of SD, rats were left in isolation under chronic light for an additional 7 days. This 7-day “rest” period after SD was adopted because PTSD typically manifests after a delayed period of time in humans. This ensured that any effects were due to long-lasting, rather than acute, effects of SD. On day 14, behavioral tests were performed, and rats were sacrificed on day 15. 

### 4.5. Drug Preparation and Administration

SDV and SDD rats were injected every other day for three weeks, beginning one week before their first day of SD, and ending at sacrifice (total of 11 injections). Rats did not receive injections on the day of sacrifice, and injections were given after behavior experiments on day 14 to avoid confounding behavior results. 

Both propranolol and mifepristone (β-adrenergic and GR antagonists) were combined and administered as single injections. While dividing rats into control, SD, SD vehicle-treated (SDV), and SD drug-treated (SDD) rats and combining antagonists did not allow for a classic 2 × 2 analysis accounting for drug and SD stress, this allowed us to minimize stress effects of repeated injections and reduce the number of animals that were used, per the IACUC’s request. We plan on exploring the individual effects of these drugs in the future. We included a vehicle control group to assess the effects of the injections alone on behavior and physiology but did not include a group that received the antagonists in the absence of SD stress. We acknowledge that this is a limitation that prevents us from examining the impact of the drugs independent of SD stress. However, while these limitations should be considered when interpreting these data, we feel that the conclusions we draw here still have merit and impact. 

Propranolol and mifepristone were ordered from Sigma-Aldrich, and saline and propylene glycol for vehicles were purchased from our university medical center and MWI, respectively. Propranolol was dissolved in sterile saline and administered at a 10 mg/kg dose, and mifepristone was dissolved in sterile propylene glycol and administered at a 10 mg/kg dose. Vehicle injections were made up of volumes of saline and propylene glycol to match the drug-injected rats from the same stress susceptible group. Drug and vehicle solutions were administered via intraperitoneal (IP) injections using sterile 25-gauge needles and syringes. 

### 4.6. Elevated plus Maze (EPM)

Avoidance of external reminders of distressing memories is one of the DSM-5 diagnostic criteria for PTSD. The elevated plus maze (EPM) has been considered the gold standard for assessing approach–avoidance behavior in rodents [83] and was used to quantify avoidance-like behaviors following SD exposure in this study. The EPM apparatus was manufactured by the BYU Precision Machining Laboratory from black plastic and had four arms: two open and two closed. The arms of the maze were 5 inches wide, and 45 inches long from the end of one arm to the end of the opposite arm. The closed arms had walls that were 18 inches high, and the open arms had no walls. During the assay, the maze was elevated 47 inches above ground on an aluminum stool. On day 14 of the stress protocol, rats were placed in a closed arm of the EPM and were observed for five minutes. The time spent in closed versus open arms and the number of times an open arm was entered were recorded. 

### 4.7. Light–Dark Transition (LDT)

The light–dark transition test (LDT) is another commonly used test in rodents to assess approach–avoidance conflict [84]. In this study, we also used the LDT to quantify avoidance-like behaviors. The light–dark transition boxes (16-inch × 16-inch × 16-inch) were manufactured by the Precision Machining Laboratory at BYU. The dark half was made out of black plastic and the light side was made out of white plastic. The boxes were put together with a removable black plastic divider separating the two sides. The divider was raised just enough to allow the rat to move between the two sides freely. The LDT boxes were placed in a dark room and an electric LED light was placed in the white box. On day 14 of stress with chronic light, the rats were placed in the dark side of the LDT boxes. Rats were observed for five minutes. The amount of time spent in dark versus light box and how many times the light box was entered were recorded. 

### 4.8. Brain Slice Preparation

To prepare brain slices, rats were first anesthetized with isoflurane, and euthanized via decapitation. The brain was rapidly removed and placed in ice-cold oxygenated artificial cerebrospinal fluid (ACSF) (in mM): NaCl, 119; NaHCO_3_, 26; KCl, 2.5; NaH_2_PO_4_, 1; CaCl_2_, 2.5; MgSO_4_, 1.3; glucose, 10; saturated with 95% O_2_, 5% CO_2_ (pH 7.4). Transverse brain slices (400 μm thick) were cut in the ice-cold oxygenated ACSF using a Leica vibratome. BLA slices were placed in an incubator at 35 °C for one hour and then moved to a submersion chamber containing oxygenated ACSF at room temperature. VH slices were placed in the submersion chamber containing oxygenated ACSF at room temperature. Brain slices acclimated by resting for at least one hour before being used in field electrophysiology experiments. Brain slice coordinates for field electrophysiology slices were adult rat bregma of dorsoventral ~−7.8 to −7.0 for LH and ~−8.5 to 8.0 for BLA. 

### 4.9. Field Electrophysiology

Following acclimation, VH slices were transferred to a submerged recording chamber in which they were perfused with oxygenated ACSF between 30 and 32 °C. BLA slices were transferred to an interphase recording chamber and were perfused with oxygenated ACSF containing 100 µM picrotoxin at 32 °C. Slices were continuously perfused with oxygenated ACSF at a flow rate of 2–3 mL/min for submerged and 1 mL/min for interphase chambers. A bipolar stainless-steel stimulating electrode was placed in the stratum radiatum CA1 of the VH or the external capsule of the BLA. Borosilicate glass patch pipettes (2–3 MΩ) were filled with 1 M NaCl for field recording electrodes and were placed ~400–700 μm from the stimulating electrode. Stimulation occurred at 80–300 µA for 100 μsec at 0.1 Hz. Recordings were performed in current clamp to measure excitatory postsynaptic potentials (EPSPs) using an Axopatch 200B or 700B amplifier. Stimulation intensity was adjusted to elicit an EPSP of amplitude 0.5–0.8 mV for VH and 0.3–1.6 mV for BLA at the beginning of each experiment. Theta burst (TB) stimuli (2 × 10 pulses at 100 Hz with 200 ms between stimuli) was the conditioning stimulus used to induce LTP in the hippocampal slices. EPSPs were evoked and monitored for at least 60 min post-conditioning at a stimulus of 0.1 Hz. Data were filtered at 4 kHz and acquired with an axon 1440A digitizer (Molecular Devices, San Jose, CA, USA) and inputted into computers with pClampClampex software version 10.4 (Molecular Devices). Only the first monosynaptic EPSC response was measured and used for data analysis. The EPSPs slopes for VH experiments and amplitude of EPSPs for BLA experiments were calculated using pClampClampfit software version 10.4 (Molecular Devices). EPSPs were measured every 10 s and were averaged into 1 min intervals. Values were normalized to pre-theta burst baseline recordings 10–15 min immediately prior to conditioning. Normalized EPSP slope (VH) and amplitude (BLA) values were compared at 25–29 min, 45–49 min, and 85–89 min post-HFS. A significant increase in EPSP slope or amplitude values that persisted longer than 30 min indicated that LTP was induced. Only one experiment was performed per slice and the reported “n” is the number of rats, with one slice used per rat included in the data sets. Microsoft Excel, IBM SPSS, Origin (Natwick, MA, USA).

### 4.10. Tissue Extraction and Reverse Transcription Quantitative PCR (RT-qPCR) 

Bilateral tissue punch samples were acquired using a dissecting scope of visually identified VH and BLA at bregma, anteroposterior −5.0, mediolateral 5.0, dorsolateral −7.2, and anteroposterior −2.5, mediolateral 4.5, dorsolateral −8.3, respectively, were obtained from 400 µm thick brain slices that were placed in filtered, oxygenated ACSF during the extraction. Tissue was homogenized, and the mRNA was extracted using TriZOL (Invitrogen, Carlsbad, CA, USA) per the manufacturer’s instructions. After extraction, reverse transcription was performed on each mRNA sample according to the iScript Supermix (BioRad, Hercules, CA, USA) protocol to create a cDNA library for each. This mixture was then cycled in a C1000 Thermocycler (BioRad) according to the iScript reaction protocol: 25.0 °C for 8 min, 42.0 °C for 60 min, and 70 °C for 15 min. Efficacy of each step was measured for both quantity and purity using a spectrophotometer.

For quantitative PCR, cDNA from the iScript reverse transcriptase reaction described above was used. The following 15 targets were investigated: β1 adrenergic receptor (β1-AR), β2 adrenergic receptor (β2-AR), α1 adrenergic receptor D (α1D-AR), glucocorticoid receptors (GR), mineralocorticoid receptors (MR), glutamate AMPA receptor 1 (AMPA1), glutamate AMPA receptor 2 (AMPA2), glutamate NMDA receptor 2A (NMDA2A), glutamate NMDA receptor 2B (NMDA2B), period circadian regulator 1 (PER1), early growth response 1 (EGR1), histone deacetylase 3 (HDAC3), CREB binding protein (CREBBP), catechol-O-methyltransferase (COMT), and nuclear receptor 4A2 (NR4A2). Adrenergic and corticosteroid receptors are important for stress hormone signaling; glutamate AMPA and NMDA receptors are essential for excitatory synapse plasticity; PER1, EGR1, HDAC3, CREBBP, and NR4A2 are epigenetic modifiers that influence LTP and memory. For example, the repressive histone deacetylase HDAC3 and the circadian gene Per1 differentially affect long-term memory in the hippocampus by restricting or promoting its formation [85]. CREBBP is a histone acetyltransferase that when genetically knocked out can impair hippocampal LTP and contextual fear [86]. Lastly, NR4A2 is a transcription factor that is critical for transcript-dependent LTP, in which NR4A2 heterozygous mice showed reduced ability to form long-term emotional memories [87]. COMT is involved in degrading synaptic dopamine, which modulates synaptic plasticity [88]. COMT genes are regulated by DNA methylation and polymorphisms of COMT have been implicated in susceptibility to PTSD [89].

Each target was run individually in triplicate and cycle threshold (Ct) was determined for each by their average. Each sample was run on a CFX96 qPCR machine (BioRad) using Sso Fast EvaGreen Supermix (BioRad) according to the following protocol: 95 °C hot start for 3 min, followed by 50 cycles of 95 °C for 15 s, 57 °C for 20 s, and 72 °C for 25 s. Amplification was measured using FAM (excitation at 488 nm, absorption at 494 nm, and emission at 518 nm) by detecting increased relative fluorescence during each cycle. A Ct value was assigned to each target using BioRad CFX Manager Software. The 18S ribosomal gene was used as a housekeeping control for expression comparison, except for PER1, EGR1, HDAC3, CREBBP, and NR4A2 where 18S and glyceraldehyde 3-phosphate dehydrogenase (GAPDH) were used and averaged together to normalize data between preparations. All the forward and reverse primers besides 18S were purchased from realtimeprimers.com and Invitrogen, the sequences for which can be seen in Table 1. Microsoft Excel and the Livak and Schmittgen delta delta Ct/Cq method [90] were used to determine the relative quantities of gene expression and GraphPad Prism was used to organize and graph data. For each individual target, all four treatment groups were run on the same plate for more accurate comparisons. Samples were eliminated if less than two of the three replicates appeared, resulting in varying sample sizes for the different targets. See Table 1 for a complete list of target genes.

### 4.11. Competitive Enzyme-Linked Immunosorbent Assay (ELISA)

To determine differences in plasma corticosterone and norepinephrine levels, corticosterone (MBS761865), norepinephrine (MBS760375), and ACTH (MBS453311) ELISA kits were purchased from MyBioSource (San Diego, CA, USA). Whole blood was collected between 9–11:30 AM after euthanasia and subsequent slices were used for electrophysiology and RT-qPCR. For each rat, 1 mL of blood was collected with 200 µL of heparin to prevent clotting. The blood samples were stored at −80 °C until the ELISAs were performed. The blood samples were brought to room temperature and processed to isolate blood plasma. The ELISAs were performed according to the manufacturer’s instructions and the plates were read at a 450 nm optical density on a Victor Nivo Multi-Mode microplate reader. Standard curves were then plotted in Microsoft Excel and used to determine the corticosterone, norepinephrine, and ACTH concentrations.

### 4.12. Statistics

One-way ANOVAs with Tukey HSD post hoc analyses were performed to determine statistical significance for all behavioral assays, RT-qPCR, and ELISAs. Two-way ANOVAs were used to determine statistical significance for field electrophysiology with Bonferroni post hoc correction for SPS data and Tukey HSD post hoc tests for SD data. Statistical significance was designated as *p* < 0.05 and a trend as *p* < 0.10. All data were checked for normal distribution before performing statistical analysis in GraphPad Prism. All analyses and figure production were performed in GraphPad Prism.

## 5. Conclusions

An obstacle in conducting PTSD research has been the difficulty in developing animal models that accurately reproduce PTSD-like symptoms observed in humans. Therefore, there are inherent limitations in studies that use animal models of the disease. Although PTSD is a neuropsychiatric disorder classified by symptoms undetectable in rodents (e.g., flashbacks, nightmares, etc.), together our behavioral, electrophysiological, and gene expression data suggest that our SD stress protocol was effective in inducing PTSD-like anxiety and stress, and that antagonizing ß-adrenergic and glucocorticoid receptors with propranolol and mifepristone was effective in mitigating some of these stress-related effects. Currently, psychotherapy is the most common and effective form of treatment for PTSD patients. However, while most patients improve following psychotherapy, the majority of patients continue to have substantial residual symptoms [91]. Overall, our data show that the SD stress protocol was effective in inducing elevations in plasma corticosterone, behavioral anxiety, VH, and BLA LTP, and alterations in gene expression. We demonstrate that propranolol and mifepristone may have potential as a prophylactic treatment in preventing SD-induced stress effects in rodents. However, future research will be necessary to determine their efficacy in humans in reducing the risk of developing PTSD and improving quality-of-life post-trauma.

## Figures and Tables

**Table 1:** DNA primer sequences of post-chronic stress-related target genes. Gene name, protein name, amplicon length in base pairs, and forward and reverse primer sequences are provided in the table.

Gene	Protein Name	Amplicon	Forward Sequence	Reverse Sequence
*ADRB1*	Beta adrenoceptor 1	219	ATCGTGCTGCTCATCGTAGT	TAGCACGTCTACCGAAGTCC
*ADRB2*	Beta adrenoceptor 2	209	GAGCACAAAGCCCTCAAGAC	TGGAAGGCAATCCTGAAATC
*ADRA1D*	Alpha adrenoceptor 1D	135	GGAAAAGATCCGTGGACAGT	AGCGGAAGAGCAACAGATTT
*NR3C1*	nuclear receptor 3C1	192	GCTTCAGGATGTCATTACGG	TCGAGCTTCAAGGTTCATTC
*NR3C2*	nuclear receptor 3C2	109	ACGCTGTGAGACTGGATTTC	AGTTACCCGGAGACACATGA
*GRIA1*	AMPA receptor subunit 1	157	CAAGGAACTGCAGGAAGAAA	CTAGAAAACCGGTGCAGAAA
*GRIA2*	AMPA receptor subunit 2	109	GAGGAAGAAAGGGAAACGAG	TCAGTCCCCATAAAACAGGA
*GRIN2a*	NMDA receptor subunit 2A	278	GCTGTCAGCACTGAATCCAA	GCCATTGACCGTTTGAAGTT
*GRIN2b*	NMDA receptor subunit 2B	194	CATCGTCACCACCTACTTCC	CCTTCGTGCAATAAAGGAGA
*PER1*	Period circadian regulator 1	60	AGAACAAGGTGGGAGCTCTT	TAGCTGGTGCCATTCTCTTC
*EGR1*	Early growth response 1	100	CGCTCACTCCACTATCCACT	GGTTTGATGAGTTGGGACTG
*HDAC3*	Histone deacetylase 3	211	CCCCCTTTCCTCAAACTCTC	TTGCATGGAAGCAAGAACTG
*CREBBP*	CREB binding protein	60	CAGAGACAAGCACTGGGAGT	ATGCACAGAGTGGACCATTT
*COMT*	Catechol-O-methyltransferase	145	AATGTCCAGACGCCAAATAA	CTGGATACTGGGGATGACAG
*NR4A2*	Nuclear receptor 4A2	61	TCTCCTGACTGGCTCTATGG	AGCAAAGCCAGGAATCTTCT

## Data Availability

The data presented in this study are available on request from the corresponding author.
